# Quantitative electroencephalographic analysis of delirium tremens development following alcohol‐withdrawal seizure based on a small number of male cases

**DOI:** 10.1002/brb3.2804

**Published:** 2022-10-28

**Authors:** Jee‐Eun Yoon, Heejung Mo, Dong Wook Kim, Hee‐Jin Im

**Affiliations:** ^1^ Department of Neurology Uijeongbu Eulji Medical Center, Eulji University School of Medicine Uijeongbu Republic of Korea; ^2^ Department of Neurology, Dongtan Sacred Heart Hospital Hallym University College of Medicine Hwaseong Republic of Korea; ^3^ Department of Neurology School of Medicine, Konkuk University Seoul Republic of Korea

**Keywords:** alcohol‐withdrawal seizure, delirium tremens, quantitative EEG, spectral analysis

## Abstract

**Introduction:**

Seizures and delirium tremens (DTs) are recognized as severe alcohol‐withdrawal symptoms. Prolonged admission and serious complications associated with alcohol withdrawal are responsible for increased costs and use of medical and social resources. This study investigated the predictive value of quantitative electroencephalography (QEEG) for developing alcohol‐related DTs after alcohol‐withdrawal seizure (AWS).

**Methods:**

We compared differences in QEEG in patients after AWS (*n* = 13). QEEG was performed in the intensive care unit within 48 h of admission, including in age‐ and sex‐matched healthy controls. We also investigated the prognostic value of QEEG for the development of alcohol DTs after AWS in a retrospective, case‒control study. The spectral power of each band frequency and the ratio of the theta to alpha band (TAR) in the electroencephalogram were analyzed using iSyncBrain^®^ (iMediSync, Inc., Korea).

**Results:**

The beta frequency and the alpha frequency band power were significantly higher and lower, respectively, in patients than in age‐ and sex‐matched healthy controls. In AWS patients with DTs, the relative beta‐3 power was lower, particularly in the left frontal area, and the TAR was significantly higher in the central channel than in those without DTs.

**Conclusion:**

Quantitative EEG showed neuronal excitability and decreased cognitive activities characteristic of AWS associated with alcohol‐withdrawal state, and we demonstrated that quantitative EEG might be a helpful tool for detecting patients at a high risk of developing DTs during an alcohol‐dependence period.

## Graphical abstract

Considering the complications related to alcohol withdrawal syndrome (AWS) such as seizure, delirium tremens and cardiac arrhythmia, it is important to identify the risk of delirium tremens (DT) following AWS and enhance preventive strategies. We observed the characteristics of quantitative electroencephalography in patients with AWS, specifically who developed DT.

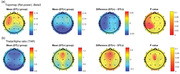

## 1 INTRODUCTION

Alcohol‐withdrawal seizures (AWS) occur in alcohol‐dependent individuals, typically 1‒3 days after the last drink, peak at about 48 h, and are markedly reduced by days 5‒7 of abstinence (Hillbom et al., 2003). As a rebound phenomenon, AWS are linked to the abrupt cessation of prolonged intoxication and alcohol abuse. Alcohol acts on the brain via several mechanisms. During alcohol abstinence, NMDA receptor function is enhanced, GABAergic transmission is reduced, and the dopaminergic system is dysregulated (Nutt, 1999). Disruption of neurochemical balance has been documented in alcohol‐withdrawal syndrome, such as higher glutamate (one of the main excitatory neurotransmitters) and lower GABA in patients with delirium tremens (DTs) than in nonalcoholic controls (Aliyev & Aliyeve, 2002), and an increase of glutamate levels in the nucleus accumbens, which is linked to addiction (Bauer et al., 2013). The enhanced excitatory neurochemical response during alcohol abstinence leads to withdrawal symptoms and signs.

Alcohol‐withdrawal symptoms include tremor, insomnia, nausea or vomiting, transient hallucinations or illusions, psychomotor agitation, anxiety, and seizures. Seizures and delirium are recognized as severe forms of alcohol‐withdrawal symptoms. Previous reviews showed that 4–15% of people with alcohol dependency (and up to 20–30% in admitted patients) develop DTs or seizures (Mennecier et al., 2008). Withdrawal seizures may occur 12–48 h after the last drink, and individuals may subsequently develop DTs within 48–72 h (Mainerova et al., 2015). DTs, also called withdrawal delirium, is an abrupt onset of fluctuating confusion, disorientation, and encephalopathic conditions, including hallucinations. It is characterized by severe hyperadrenergic and dysautonomic states, including hyperthermia, cardiac arrhythmias, complications of withdrawal seizures, or concomitant medical disorders, which can lead to death in approximately 1–4 % of hospitalized patients (Mainerova et al., 2015). The mortality in the 8 years following an episode of DTs is 30.8% (hazard ratio 1.38, 95% confidence interval 0.43–4.48), which is comparable to that in patients with severe malignant diseases (Coleman et al., 2008).

Clinically significant predictors for the occurrence of DTs include, a history of DTs, lower serum potassium, lower platelet count, presence of structural brain lesions, and a high pulse rate above 100–120 bpm (Eyer et al., 2011; Lee et al., 2005). In previous studies, admission with seizures per se and older age (>70 years) were found to increase the risk of hallucinations and delirium as a withdrawal complication in general hospitals. The biggest hazard for delirium is an increased number of days since the last drink (Ferguson et al., 1996). However, previous studies have mainly focused on predicting the severity of alcohol‐withdrawal symptoms, particularly seizures, and differed in their methodology. Thus, there is no general consensus on which factors increase the risk of DTs.

A predictive tool for the risk of DTs development after AWS would assist clinicians in making therapeutic decisions and thereby reduce patient mortality as well as the socioeconomic burden associated with alcoholism. Quantitative electroencephalography (QEEG) allows the analysis of quantitative features of brain function via oscillatory electrophysiological rhythms, such as spectral power and coherence. Various reports have recently examined the role of QEEG as a potential neurodynamic biomarker that helps in detecting and predicting neurological disorders such as Alzheimer's disease (AD), Parkinson's disease dementia, and Huntington's disease (Poil et al., 2013; Klassen et al., 2011; Odish et al., 2018). For example, beta frequency band (13–30 Hz) could predict progression to AD at the mild cognitive impairment stage (Poil et al., 2013).

QEEG studies of alcoholics have reported decreased alpha power, increased delta, theta, and beta power, and low‐voltage fast desynchronized pattern (Funderburk, 1949; Courtney & Polich, 2010). However, the majority of QEEG investigations in alcohol use disorder have recruited the participants from outpatient treatment facilities, showing the neurophysiologic effects of chronic alcohol exposure rather than the acute phase of alcohol withdrawal. A few studies on delirium detection using QEEG showed significantly increased theta and delta activities, and decreased alpha activity in delirium patients (Van Der Kooi et al., 2015). Relative delta power in the frontal‒parietal electrode pair differ between patients with and without delirium who underwent cardiothoracic surgery (Van Der Kooi et al., 2015). Considering that QEEG might reflect brain dysregulation by the altered balance of excitatory/inhibitory neurotransmitters during AWS, QEEG may be a useful tool to detect alcohol‐withdrawal syndrome and predict the development of alcohol DTs with the advantages of noninvasiveness, portability, and low cost. In this study, we aimed to evaluate the differences in QEEG between patients who have had AWS and healthy controls, and to investigate early QEEG changes for the development of alcohol‐related DTs after AWS.

## 2 MATERIALS AND METHODS

### 2.1 Participants

We retrospectively reviewed the electronic medical records of all patients who were diagnosed with AWS at admission to two university hospitals (Hallym University Hangang and Dongtan Sacred Heart Hospital) between March 2018 and December 2020. We enrolled 38 patients aged 19 years or older who underwent initial neuroimaging, such as computed tomography and/or magnetic resonance imaging, and EEG, within 48 h after seizure, in the intensive care unit. Patients were classified into two groups, according to whether they developed DTs or not after admission. The diagnosis of alcohol‐withdrawal syndrome and DTs was based on the Diagnostic and Statistical Manual of Mental Disorders (DSM‐5) (Association, 2013). AWS is defined as an episode of seizure occurring within 1‒5 days after abrupt cessation of alcohol in patients with alcohol‐withdrawal syndrome. We excluded patients with structural brain lesions on brain imaging that could confound the EEG findings (Duncan, 1997) and those in whom it was not possible to evaluate DTs due to underlying language disturbance, hearing difficulty, or cognitive impairment. After excluding 25 patients, 13 patients were finally analyzed.

For the comparison of EEG between the healthy controls and patients, we selected data of age‐ and sex‐matched healthy adults (*n* = 13) from the big data collected during the development of the EEG spectral analysis software iSyncBrain® (iMediSync, Inc., Korea) (Ko et al., 2021). Briefly, QEEG data from healthy volunteers were collected between 2014 and 2019 to provide the range of normative values of QEEG to control for the effects of age and sex. A total of 1,289 participants (aged 4.5 to 81 years; men 43.1%) from a community based population were recruited by a two‐step screening procedure. Step 1 consisted of a structured telephone interview with a questionnaire designed to exclude participants who have any history of medical (such as cardiac, pulmonary, and renal disease, or metabolic syndrome), neurological (such as stroke, epilepsy, or head trauma), psychological illness (such as behavioral or conduct disorders). The step 1 prescreening interview was terminated when one of the above exclusion criteria was met. Step 2 consisted of a face‐to‐face interview which was performed at the Korean EEG Center at Seoul National University Hospital for assessing cognitive function, depressive mood, and anxiety by using a structured questionnaire. The inclusion criteria of the normal control group were as follows: (1) individuals from a community based population; (2) no history or symptoms of medical, neurological, and psychiatric illness; (3) no history or symptoms of cognitive impairment; (4) a Mini‐Mental State Examination score > 23; (5) a Korean‐Instrumental Activities of Daily Living ≤ 0.42; (6) a Korean Dementia Screening Questionnaire score ≤ 6; (7) ≥6 years of education; (8) Beck Depression Inventory score ≤ 9; (9) State‐Trait Anxiety Inventory Korean YZ Form ≤ 55. The informed consents were obtained from all subjects. This study was approved by the Institutional Review Board (IRB) of Hallym University Medical Center (IRB No. HG2019‐027 and DT2021‐018). All methods were performed in accordance with the relevant guidelines and regulations

### 2.2 Clinical assessment

Clinical variables included age, sex, amount of alcohol consumption, and duration of alcohol intake for all patients. Patients were asked questions on the frequency (days per week), quantity (standard unit per unit of time), and types of beverages such as soju (Korean traditional alcohol), beer, and wine, frequency (number of days per week), and quantity (number of standard unit each time) of alcohol consumption. A standard unit was defined as a bottle for each type of alcohol. Generally, one bottle of soju (360 ml) and one bottle of beer (330 ml) contain 72 g and 12 g of ethanol, respectively. The level of alcohol consumption was calculated as mean ethanol intake per day with the following formula: (frequency of consumption per week x number of standard units each time x amount of ethanol)/7. After admission to the ICU, blood samples were collected for assessment of white blood cell count, mean corpuscular volume, platelet count, prothrombin time and hemoglobin, albumin, vitamin B12, folic acid, aspartic acid transaminase, alanine aminotransferase, total bilirubin, direct bilirubin, γ‐glutamyl transferase, blood nitrogen urea, total cholesterol, ammonia, prolactin, homocysteine, and neuron‐specific enolase levels.

### 2.3 Electroencephalogram recording

All patients underwent EEG recording within 48 h after clinical seizure. Electrodes were placed according to the International 10–20 System. Patients were verbally instructed to lie down, relax all their muscles, specially the cranial muscles, and minimize blinking and eye movements. Nineteen channels, with a common average reference montage, were used for recording, that is, Fp1, F3, C3, P3, Fp2, F4, C4, P4, F7, T3, T5, O1, F8, T4, T6, O2, Fz, Cz, and Pz. Data were sampled with a frequency of 250 Hz and the acquired signals were filtered with digital high‐and low‐pass filters at 0.3 and 70 Hz, respectively. EEG recordings were conducted for at least 20 min, of which at least 5 min of awake EEG were included for each patient. The circumstances for recording and sources of artifacts were identical for all patients and were controlled during the examination in the intensive care unit. Two neurologists (H. Mo and H‐J Im) selected 5 min of continuous eye‐closed and artifact‐free awake EEG data for further analysis.

### 2.4 Data processing and analyses

All preprocessing and data analyses were implemented using EEGLAB software. The selected EEG data were high‐pass filtered above 1 Hz, low‐pass filtered below 45 Hz, and recomputed to obtain a common average reference, offline. In addition to visual inspection, using advanced mixture‐independent component analysis (amICA), transient and stationary artifacts originating from eye movement, muscle, or heartbeat were removed. Fast Fourier transform was used to calculate the spectral band power with a Hanning window (4‐s sliding window with 50% overlap). The spectral frequency bands were categorized into the eight categories: δ (1–4 Hz), θ (4–8 Hz), α1 (8–10 Hz), α2 (10–12 Hz), β1 (12–15 Hz), β2 (15–20 Hz), β3 (20–30 Hz), and γ (30–45 Hz) (Delorme & Makeig, 2004; Delorme et al., 2012). The absolute and relative power of each frequency band were calculated.

**FIGURE 1:**
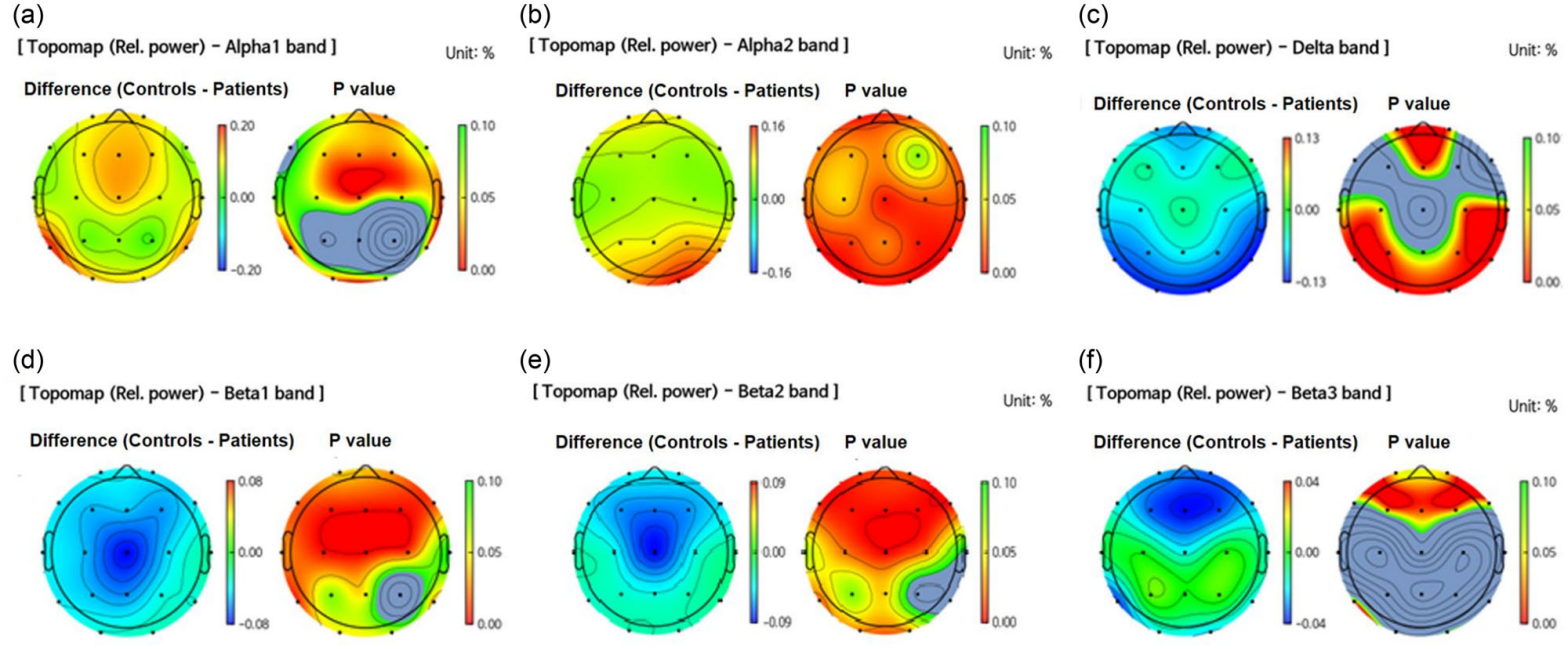
**Topomap of comparison of relative spectral power bands between the patients after AWS and healthy controls**: (a) alpha‐1 band (8–10 Hz), (b) alpha‐2 band (10–12 Hz), (c) delta band (1–4 Hz), (d) beta‐1 band (12–15 Hz), (e) beta‐2 band (15–20 Hz), (f) beta‐3 band (20–30 Hz). AWS, alcohol‐withdrawal seizure.

**FIGURE 2:**
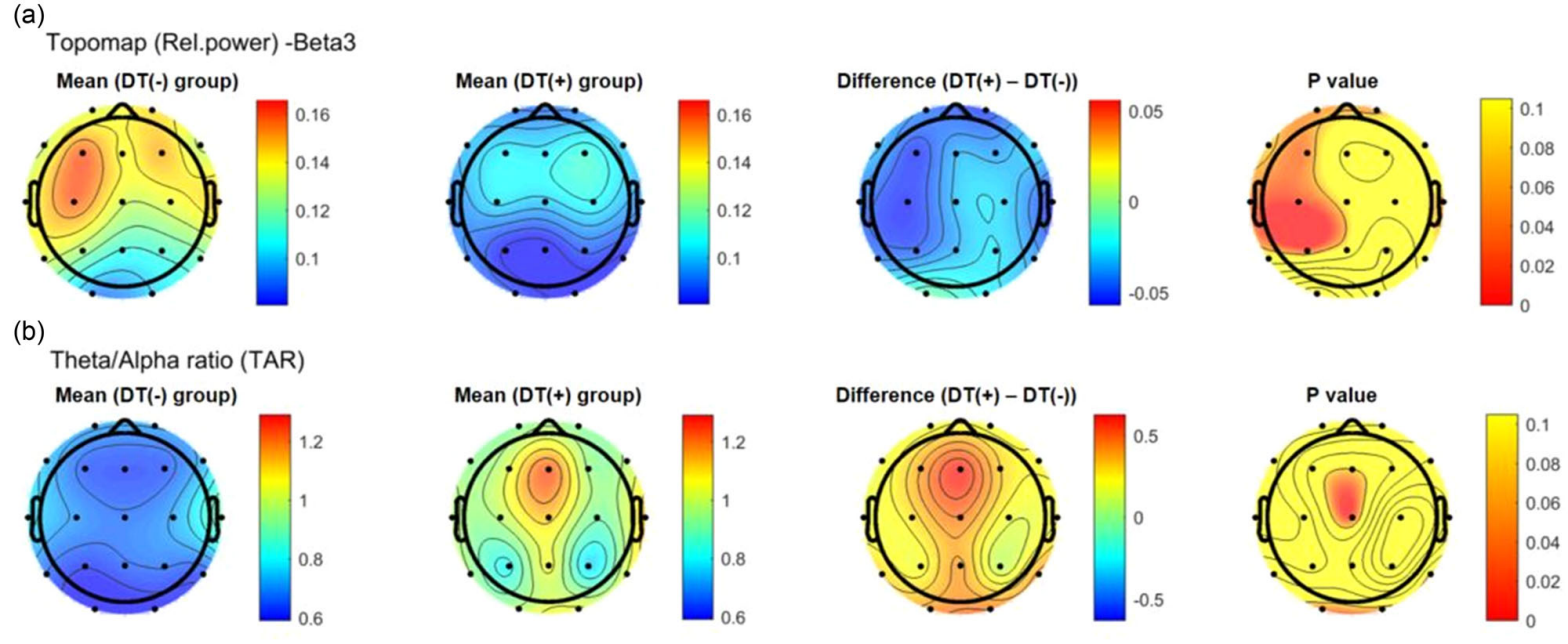
**Topomap of comparison of relative spectral power band and theta/alpha band ratio between the patients with and without delirium tremens**: (a) beta‐3 band (20–30 Hz), (b) theta/alpha band power ratio. DTs, delirium tremens, +, with; –, without.

All the preprocessing steps, de‐noising using amICA, sensor‐level feature extraction, source‐level feature extraction, and generation of topomap images were performed on iSyncBrain ® (iMediSync, Inc., Seoul, Korea) (https://isyncbrain.com/). The color scale bar indicates the difference between the group of interest and the other group. In Figure 1, a topomap shows the differences between the patients and controls, with colors toward the red end of the spectrum indicating that the group of interest (the patient group) had a relatively lower power than the control group. Conversely, colors toward the end blue end of the spectrum indicate higher power. In Figure 2, a topomap shows the differences between the DTs (–) and DTs (+) group, with colors toward the red end of the spectrum indicating that the group of interest (DTs(+)) had a relatively higher power than the other group (DTs(–)). Conversely, colors toward the blue end of the spectrum indicate lower power. *p* Values toward the red end indicate statistical significance (*p* < .05), whereas *p* values toward the green end indicate nonsignificant results (*p* > .05). The analysis protocol has been documented in previous reports (Baik et al., 2021).

### 2.5 Statistical analysis

Continuous variables with normal distributions are presented as mean ± standard deviation, while those with nonnormal distributions are presented as medians (interquartile ranges). We compared the baseline demographic information and risk factor profiles of patients with DTs. We used the Mann‒Whitney *U* test for comparison of continuous variables and Fisher's exact test for comparison of categorical variables. All statistical analyses in the current study were performed using SPSS (version 24.0; IBM SPSS, Chicago, IL, USA). Statistical analyses of the EEG features were performed using MATLAB (R2017b, MathWorks, Inc.). For each band, a Mann–Whitney test was performed on absolute and relative power within that band according to each channel, testing the AWS group vs. normal controls and DTs patients vs. non‐DTs patients. Statistical analysis was performed automatically using the iSyncBrain® program.

## 3 RESULTS

### 3.1 Demographic and clinical characteristics of patients

Consecutive patients who visited the emergency department between March 2018 and December 2020 for acute seizures, deemed to be cases of alcohol‐withdrawal by a physician, were retrospectively included in the study. Thirteen patients were finally enrolled in the study. The mean age of participants was 50.1 ± 10.7 years, and all patients were male. DTs occurred in eight of the 13 patients (61.5%). Most of them (10 of 13 patients, 76.9%) experienced their first‐ever seizure at admission; for the others, all previous seizures occurred during the alcohol‐withdrawal state. AWS occurred on average 31.5 ± 21.5 h after the self‐reported last drinking episode. The average daily amount of alcohol consumption was 160.1 ± 65.9 g/day.

The demographics and clinical characteristics of the patients with and without DTs are shown in Table 1. There were no significant differences in demographic and clinical alcohol‐related factors, such as age, sex, history of psychiatric disorders and AWS, time interval from the last drink to seizure, and average amount of alcohol consumed per day between the two groups. The results of laboratory tests and use of benzodiazepines and antiseizure medication for the treatment of AWS also did not show statistically significant differences between the groups. The duration of alcohol intake across life (in year) was longer in the patients with DTs than in the patients without DTs.

**TABLE 1:** Demographic and clinical characteristics of patients with and without delirium tremens

Characteristics	DTs (+) (*N* = 8)	DTs (–) (*N* = 5)	*p* Value
Age (years)	47.6 ± 10.7	54.0± 10.5	.061
Male	8 (100.0)	5 (100.0)	.692
Platelets (g/L)	139.8 ± 86.0	137.2 ± 59.6	.943
γ‐GT (mmol/L)	436.0 ± 364.5	1056.6 ± 481.7	.065
Na+ (mmol/L)	138.0 ± 3.7	138.8 ± 3.6	.943
K+ (mmol/L)	3.9 ± 0.4	3.5 ± 0.8	.185
BUN (mg/dl)	10.0 ± 0.4	6.8 ± 2.8	.107
Creatinine (mg/dl)	0.9 ± 0.2	0.9 ± 0.1	.622
Homocysteine (umol/L)	21.6 ± 10.4	44.8 ± 7.4	.111
Benzodiazepine	8 (100)	4 (80)	.385
Antiseizure medication	2 (25)	0 (0)	.487
Psychiatric disorders	2 (25)	2 (40)	1.000
Previous alcohol‐withdrawal seizures (Yes)	3 (37.5)	0 (0)	.581
Last drink before seizure			.762
Less than 1 h	3 (37.5)	1 (20.0)	
24–72 h	4 (50.0)	3 (60.0)	
More than 72 h	1 (12.5)	1 (20.0)	
Amount of alcohol consumed/day (g/day)	163.0 ± 78.0	155.5 ± 48.2	.883
Duration of alcohol intake across life (year)	17.1 ± 5.4	32.0 ± 10.4	.011*

*Note*: The data are presented as the mean ± the standard deviation or as the number (%).

*p < .05, based on the Mann–Whitney *U* test or Fisher test.

γ‐GT, γ‐glutamyl transferase; BUN, blood nitrogen urea; DTs, delirium tremens.

### 3.2 Comparison of spectral power pattern between alcohol‐withdrawal patients and healthy controls

We performed an absolute and relative sensor‐level analysis between the AWS patients and the age‐ and sex‐matched healthy adult group. In the absolute spectral power analysis, the AWS patients showed higher beta‐1, beta‐2, and beta‐3 power in the central (Cz) area than the healthy controls (*p* < .05). In the relative power analysis, alpha‐1 power in the virtually entire cortex, except for the parietal area, was lower in the AWS group than in the healthy control group (Figure 1a, frontal, 10.8 ± 6.6 vs. 20.6 ± 14.1, *p* < .03; central, 11.1 ± 7.8 vs. 21.0 ± 13.9, *p* = .03; temporal, 11.7 ± 8.1 vs. 24.3 ± 14.8, *p* = .01; occipital, 14.3 ± 9.5 vs. 26.5 ± 16.2, *p* = .03). The alpha‐2 power in the entire cortex was also lower in the AWS group than in the healthy control group (Figure 1b, frontal, 8.8 ± 4.2 vs. 15.3 ± 8.0, *p* = .02; central, 8.6 ± 3.0 vs. 13.9 ± 6.0, *p* = .01; temporal, 8.3 ± 2.9 vs. 15.0 ± 5.5, *p* < .01, parietal, 9.4 ± 4.5 vs. 18.6 ± 10.7, *p* < .01, occipital, 9.7 ± 4.7 vs. 23.7 ± 10.2, *p* < .01). In contrast, the AWS group showed a higher relative delta power in in the virtually entire cortex (the frontal and temporo‐parieto‐occipital areas) than normal controls (Figure 1c, frontal, 26.7 ± 7.6 vs. 20.9 ± 7.5, *p* = .04; temporal, 26.3 ± 9.7 vs. 15.8 ± 5.5, *p* < .01; parietal, 25.4 ± 8.8 vs. 17.6 ± 6.5, *p* = .02; occipital, 25.8 ± 8.2 vs. 13.1 ± 5.5, *p* < .01). All range of beta power (beta‐1, beta‐2, and beta‐3) mostly in the frontal and central areas were higher in the AWS group than in the normal controls (Figure 1d–f, beta‐1: frontal, 10.2 ± 4.9 vs. 5.7 ± 2.5, *p* = .01; central, 11.7 ± 4.7 vs. 6.1 ± 2.6, *p* < .01, beta‐2: frontal, 12.0 ± 5.2 vs. 6.4 ± 2.5, *p* < .01; central 13.9 ± 5.6 vs. 8.3 ± 3.4, *p* < .01, beta‐3: frontal, 11.6 ± 3.9 vs. 8.4 ± 2.6, *p* = .02; central, 11.9 ± 3.5 vs. 11.3 ± 4.3, *p* = .71). Theta and gamma power did not differ between the two groups.

### 3.3 Comparison of spectral power pattern between the patients with and without DTs

To identify the difference in the spectral power pattern and find early predictive features of DTs, we compared the QEEG performed immediately (within 48 h) after AWS between the groups of participants with (*n* = 8) and without DTs (*n* = 5). In the absolute spectral power analysis, high‐frequency power, particularly beta‐3 power in the left frontal (Fp1 and F3) and parietal (Pz) areas, was relatively lower in patients with DTs than in those without DTs (*p* < .05). The relative beta‐3 powers of the left cortical areas (F3, T3, C3, and P3) were lower in the group with DTs than in the group without DTs (Figure 2a, F3: 0.11 ± 0.05 vs. 0.16 ± 0.03, *p* = .04; T3: 0.09 ± 0.03 vs. 0.13 ± 0.03, *p* = .04; C3: 0.10 ± 0.03 vs. 0.16 ± 0.04, *p* = .01; P3: 0.08 ± 0.03 vs. 0.13 ± 0.03, *p* = .03). However, the beta‐3 power of the right hemisphere and other ranges of spectral powers involving delta, theta, alpha, and gamma activities were not significantly different between the two groups. In the spectral power ratios, the central‐occipital) theta to alpha ratios (TAR) of DTs patients were higher than those of non‐DTs patients (central, 1.12 ± 0.38 vs. 0.67 ± 0.18, *p* = .02; occipital, 0.98 ± 0.36 vs. 0.56 ± 0.22, *p* = .04) (Figure 2b).

## 4 DISCUSSION

In this study, we compared the relative and absolute spectral power patterns across frequency bands between AWS patients in the acute period (within 48 h after AWS) and age‐ and sex‐matched healthy controls. In addition, we investigated whether spectral characteristics from early standard EEG are helpful for identifying patients at risk of developing DTs. The main findings of the current study were as follows: (1) Clinical and alcohol‐related variables were comparable between AWS patients with or without DTs. (2) In the QEEG analysis, AWS patients showed higher relative delta and all range of beta power than did the healthy control group, whereas alpha power was lower in the AWS group. The spectral differences between the groups were predominant in the frontal area (except alpha‐2 power, which was lower in almost all brain areas). (3) The absolute and relative spectral analysis showed that the high‐frequency beta band power, specifically beta‐3 (20–30 Hz), was lower in patients with than in patients without DTs. These differences were recorded with a certain topographic dominance in the left frontal cortical areas. Additionally, in terms of the spectral power ratios, patients with DTs had higher TAR than those without DTs in the central area.

Many studies have identified risk factors for DTs, but the results are inconsistent. Advancing age, past history of DTs, daily heavy alcohol use, severe alcohol‐withdrawal symptoms involving high systolic blood pressure (> 150 mmHg), tachycardia (> 100 beats/min), abnormal laboratory findings including low platelets, low potassium, high homocysteine, low pyridoxine, and low magnesium have been regarded as the potential predisposing factors for DTs (Eyer et al., 2011; Ferguson et al., 1996; Lee et al., 2005). In the current study, the patients with DTs had a higher likelihood of short‐term alcohol consumption across life in year than the patients without DTs. It suggests that drinking patterns such as binge amount for short‐term and abrupt discontinuance of alcohol might play a larger role in triggering DTs rather than total duration of alcohol consumption across life. In addition, there could be memory problem in alcohol‐dependence, though we did not evaluate, and also due to relatively younger age in the group of patients with DTs than in the group of patients without DTs, leading to relatively fewer days of drinking, and a small sample size. Our observation of increased beta power and decreased alpha power in alcohol‐withdrawal patients, as compared to healthy controls, was consistent with previous findings of EEG abnormalities in the alcohol‐withdrawal state (Feige et al., 2007; Kaplan et al., 1985; Sand et al., 2002). Abnormal slow delta and theta activities and decreased alpha with low amplitude have been reported in patients with AWS (Sand et al., 2002). Furthermore, fast beta activity ranging from 24–32 Hz during REM sleep increased after acute alcohol withdrawal in alcohol‐dependent patients (Feige et al., 2007). However, more advanced research on spectral power analysis in alcohol‐withdrawal state, especially related to AWS, has not been conducted recently. Most previous studies investigating alcohol‐dependence showed EEG abnormalities, such as generalized reduction of alpha rhythm and increased power in the delta, theta, and beta activities (Funderburk, 1949). Compared with these previous results, our results expand the understanding of the association between alcohol use and EEG characteristics, considering that alcohol‐withdrawal syndromes are a part of alcohol‐dependence. Rebound hyperexcitability, driven by abrupt cessation from chronic alcohol exposure, might affect neuroelectric activity in the central nervous system. Changes in the glutamate/GABA balance take place during alcohol‐withdrawal periods (Brousse et al., 2012). In addition, increased beta power might reflect increasing cortical hyperexcitability after seizure as well as an alcohol‐withdrawal state, resulting from an imbalance between excitatory and inhibitory neurons (Roberto & Varodaya, 2017). This speculation is supported by the mechanisms for the generation of beta oscillations, which involves the balance between networks of excitatory pyramidal (AMPAergic) cells and inhibitory (GABAergic) interneurons (Whittington et al., 2000). Our findings might suggest the cumulative neurophysiological effects of alcohol consumption on the brain. Increased absolute power in the beta frequency range of 12.5–20 Hz has been observed in alcohol‐dependence, over all brain locations, but prominently in the central brain region, in a previous study (Rangaswamy et al., 2002). Another study showed that alcohol‐dependence was positively correlated with absolute high beta and gamma power (20–40 Hz) in the left fronto‐central‐parietal leads on EEG (Ehlers et al., 2010).

Alpha power has been reported to be linked to cortical arousal level and cognitive and memory performance (Başar, 2012). In particular, the slow alpha frequency (8–10 Hz; alpha‐1) is related to attentional demands, whereas the fast alpha frequency (10–12 Hz; alpha‐2) mediates semantic memory and stimulus‐related aspects (Adams et al., 1993). The frontal cortex (particularly the left inferior frontal gyrus) plays a role in supporting cognitive functions that are not only specific to language, as it has many afferent and efferent connections to all other neocortical regions (i.e., the parietal, temporal, and occipital regions), as well as to the cingulate, limbic, and basal ganglia structures (Tyler et al., 2011). Alcoholism may particularly affect frontal cognitive function. In various brain imaging studies, chronic alcohol intake resulted in reduction of regional cerebral blood flow impairment, affecting the function of cerebral tissue in the medial frontal region and decreasing tissue metabolic rates, with adverse neurophysiological effects (Adams et al., 1993; Moselhy et al., 2001). Alcohol‐dependence was associated with reduced absolute power and lower voltage of the alpha frequency in our study, which might reflect alcohol‐related attentional, stimulus‐reactive, and cognitive dysfunctions, as compared to healthy controls, which was consistent with previous results of alcohol‐dependence and electric deflection in the postseizure state (Paulucio et al., 2017). Taken together, the differential dominance in the frontal area between the AWS patients and the healthy control group can be explained by a strong connection of alcoholism to frontal lobe pathology and the postseizure state (Moselhy et al., 2001).

Studies using QEEG analysis to predict DTs, particularly in the alcohol‐withdrawal state, remain scarce. Our study highlighted that specific EEG characteristics could be a significant predictor of DTs, based on a direct comparison of EEG data obtained in the acute post‐AWS period. We found that the AWS group without DTs had higher absolute and relative power in the beta‐3 range than did the AWS group with DTs. Although we did not perform cognitive assessment, cognitive deterioration associated with DTs could also be an explanation for the lower beta power in the AWS patients with DTs, given that lower beta power has negative effects on memory processing, such as episodic memory encoding and retrieval (Nyhus, 2018). Patients with alcohol‐dependence with DTs had worse intellectual functioning, which was clearly observed in terms of attention and productive visual‐motor coordination as compared to patients without DTs (Dickov et al., 2012).

Findings regarding beta activity related to cognitive activity have been less consistent. However, the beta‐3 frequency, ranging from 20 to 30 Hz, has been specifically associated with cognitive processes such as semantic speech retrieval (Shahin et al., 2009), prosodic aspects (Weiss & Mueller, 2012), and working memory (Pavlov & Kotchoubey, 2017). It has been proposed that high‐frequency band power, not only beta‐3, but also gamma activity, is an indicator of cognitive activity and that coherent oscillations in this frequency range allow the binding of distant brain regions that are necessary to allow cognitive experience (Gross & Gotman, 1999). Visual or auditory stimulation and cognitive activities suppress brainwaves while increasing the power of the high‐frequency beta and gamma bands (Marrufo et al., 2001). Hence, the decreased high beta (specifically beta‐3) power in the DT group may reflect the cognitive dysfunction that precedes the development of DTs. This suggests that beta‐3 power could be used as a predictive factor for the development of DTs during alcohol withdrawal. Interestingly, the difference in beta power between patients with and without DTs was prominent in the left hemisphere. While the reasons for asymmetry of EEG spectral power remain unclear, our finding may be in line with studies indicating that the mirrored reduction of left and right asymmetry might depend on the specific cognitive domain, such as verbal (left) and visuospatial (right) functions (Nyhus, 2018). The frontal lobe, particularly the left frontal gyrus, is involved in functions such as creative thinking, planning of future actions, decision‐making, artistic expression, aspects of emotional behavior, and spatial working memory (Tyler et al., 2011). It has been reported that an increased high beta frequency band (22‒30 Hz) induced by an antiepileptic drug (levetiracetam) correlates with better neuropsychological measures in patients with epilepsy (Park & Kwon, 2009). Enhanced activities of the neuronal networks in the prefrontal cortex and left hippocampus also correlate with an increased beta‐3 band.

Another index used to explore the difference in EEG characteristics for predicting alcohol‐withdrawal delirium is the frequency band ratio. Our results showed an increased TAR in the central area in patients who developed DTs. The reverse metric of the TAR, the alpha/theta ratio, has been used as a marker of functional connectivity and performance enhancement, and is used to discriminate individuals with probable AD from healthy older controls. Increased frontal‐midline theta is related to learning in a task and during the performance of an attention‐demanding procedure. The theta frequency has been associated with long‐range functional interactions in working memory. The TAR is known to change with age and cognitive ability, even in healthy individuals. One study reported that the relationship between cognitive ability and the TAR was age‐dependent, and that cognitive performance at the CZ midline location predicted the TAR (Trammell et al., 2017). Another study showed that the TAR was increased relative to controls in older adults with amnesic mild cognitive impairment (Bian et al., 2014). Alpha/theta neurofeedback training has also been shown to have clinical benefits in the treatment of alcoholism and addiction (Gruzelier, 2009). An increased TAR in patients with AWS, specifically in those who progressed to DTs, might indicate addiction and preceding cognitive impairment, which was previously found to be associated with AD (Weiss & Mueller, 2012).

The current study had several limitations. First, the study sample was small, and there might have been a selection bias, since this study was performed at a single center. Unexpectedly, most of the subjects who were initially enrolled were subsequently excluded because of structural brain lesions on neuroimaging, to avoid interference on EEG. Most of our subjects had brain lesions in the cerebral cortex because alcohol intoxication is one of the strongest predictors of traumatic brain injury and a greater vascular burden (Weil et al., 2018). Second, all subjects were men, implying that it may be difficult to generalize this finding to women. Third, we did not perform a neuropsychiatric test to identify delirium. Further studies using neuropsychiatric scores and QEEG findings (e.g., cognitive deviation to the left and right cortical function or changes in degree between the groups) are required. Finally, alcohol consumption was not evaluated in the healthy controls, which could have led to confounding effects for normative QEEG characteristics. However, this study had several strengths. It was performed according to a strict protocol, under well‐controlled circumstances and time lines. Furthermore, no previous report has used QEEG to predict alcohol‐related DTs, particularly after AWS, which is the severe state of alcohol‐withdrawal. Given the preliminary nature of our study with its evident limitations (small population size consisting of male participants and single‐center setting), the authors are aware that QEEG might not yet be widely recognized as valuable biomarker for detecting DTs, and our results should be interpreted with caution. Further explorations and large controlled studies are needed to establish the role of QEEG as a potential biomarker to detect DTs based on the current study.

## 5 CONCLUSION

Considering the broader economic and health care burden and mortality related to DTs, early identification of patients at risk of developing delirium tremens is essential for early intervention to stop the cascade of negative outcomes. QEEG might be a helpful tool for detecting early changes in the development of DTs in alcohol‐withdrawal patients and could help physicians identify patients with AWS who are at high risk of developing DTs during an alcohol‐dependence period.

## AUTHOR CONTRIBUTIONS

H‐J Mo, JE Yoon, and H‐J Im contributed to the analysis and interpretation of the data and drafted the manuscript; DW Kim contributed to the data acquisition and interpretation, and revised the manuscript; JE Yoon interpreted results and revised the manuscript. H‐J Im conceptualized and designed the study, analyzed and interpreted the data, and revised the manuscript. All authors reviewed the manuscript.

## CONFLICT OF INTEREST

The authors declare no competing interests.

### PEER REVIEW

The peer review history for this article is available at: https://publons.com/publon/10.1002/brb3.2804.

## Data Availability

The data that support the findings of this study are available from the corresponding author upon reasonable request. The data are not publicly available due to ethical restrictions.

## References

[brb32804-bib-0001] Adams, K. M. , Gilman, S. , Koeppe, R. A. , Kluin, K. J. , Brunberg, J. A. , Dede, D. , Berent, S. , & Kroll, P. D. (1993). Neuropsychological deficits are correlated with frontal hypometabolism in positron emission tomography studies of older alcoholic patients. Alcoholism: Clinical and Experimental Research, 17(2), 205–210. 8488956 10.1111/j.1530-0277.1993.tb00750.x

[brb32804-bib-0002] Aliyev, N. N. , & Aliyev, Z. N. (2002). The role of amino‐acid transmitters in the pathogenesis of delirium tremens: A brief report. Journal of Studies on Alcohol, 30(2), 531–533. 10.15288/jsa.2002.63.531 12380848

[brb32804-bib-0003] Association, A. P. (2013). Diagnostic and statistical manual of mental disorders. American Psychiatric Publishing.

[brb32804-bib-0004] Baik, K. , Kim, S. M. , Jung, J. H. , Lee, Y. H. , Chung, S. J. , Yoo, H. S. , Ye, B. S. , Lee, P. H. , Sohn, Y. H. , & Kang, S. W. (2021). Donepezil for mild cognitive impairment in Parkinson's disease. Scientific reports, 11(1), 1–9. 33637811 10.1038/s41598-021-84243-4 PMC7910590

[brb32804-bib-0005] Başar, E. (2012). A review of alpha activity in integrative brain function: Fundamental physiology, sensory coding, cognition and pathology. International Journal of Psychophysiology, 86(1), 1–24. 22820267 10.1016/j.ijpsycho.2012.07.002

[brb32804-bib-0006] Bauer, J. , Pedersen, A. , Scherbaum, N. , Bening, J. , Patschke, J. , Kugel, H. , Heindel, W. , Arolt, V. , & Ohrmann, P. (2013). Craving in alcohol‐dependent patients after detoxification is related to glutamatergic dysfunction in the nucleus accumbens and the anterior cingulate cortex. Neuropsychopharmacology, 38(8), 1401–1408. 23403696 10.1038/npp.2013.45 PMC3682141

[brb32804-bib-0007] Bian, Z. , Li, Q. , Wang, L. , Lu, C. , Yin, S. , & Li, X. (2014). Relative power and coherence of EEG series are related to amnestic mild cognitive impairment in diabetes. Frontiers in Aging Neuroscience, 6, 11. 24550827 10.3389/fnagi.2014.00011 PMC3912457

[brb32804-bib-0008] Brousse, G. , Arnaud, B. , Vorspan, F. , Richard, D. , Dissard, A. , Dubois, M. , Pic, D. , Geneste, J. , Xavier, L. , & Authier, N. (2012). Alteration of glutamate/GABA balance during acute alcohol withdrawal in emergency department: A prospective analysis. Alcohol and Alcoholism, 47(5), 501–508. 22791370 10.1093/alcalc/ags078

[brb32804-bib-0009] Coleman, M. P. , Quaresma, M. , Berrino, F. , Lutz, J.‐M. , De Angelis, R. , Capocaccia, R. , Baili, P. , Rachet, B. , Gatta, G. , & Hakulinen, T. (2008). Cancer survival in five continents: A worldwide population‐based study (CONCORD). The Lancet Oncology, 9(8), 730–756. 18639491 10.1016/S1470-2045(08)70179-7

[brb32804-bib-0010] Courtney, K. E. , & Polich, J. (2010). Binge drinking effects on EEG in young adult humans. International Journal of Environmental Research and Public Health, 7(5), 2325–2336. 20623027 10.3390/ijerph7052325 PMC2898052

[brb32804-bib-0011] Delorme, A. , & Makeig, S. (2004). EEGLAB: An open source toolbox for analysis of single‐trial EEG dynamics including independent component analysis. Journal of Neuroscience Methods, 134(1), 9–21. 15102499 10.1016/j.jneumeth.2003.10.009

[brb32804-bib-0012] Delorme, A. , Palmer, J. , Onton, J. , Oostenveld, R. , & Makeig, S. (2012). Independent EEG sources are dipolar. PloS One, 7(2), e30135. 22355308 10.1371/journal.pone.0030135 PMC3280242

[brb32804-bib-0013] Dickov, A. , Vuckovic, N. , Martinovic‐Mitrovic, S. , Savkovic, I. , Dragin, D. , Dickov, V. , Mitrovic, D. , & Budisa, D. (2012). Disorder verbal memory in alcoholics after delirium tremens. European Review of Medical and Pharmacological Sciences, 16(8), 1052–1060. 22913156

[brb32804-bib-0014] Duncan, J. S. (1997). Imaging and epilepsy. Brain: A Journal of Neurology, 120(2), 339–377. 9117380 10.1093/brain/120.2.339

[brb32804-bib-0015] Ehlers, C. L. , Phillips, E. , Gizer, I. R. , Gilder, D. A. , & Wilhelmsen, K. C. (2010). EEG spectral phenotypes: Heritability and association with marijuana and alcohol dependence in an American Indian community study. Drug and Alcohol Dependence, 106(2‐3), 101–110. 19748744 10.1016/j.drugalcdep.2009.07.024 PMC2815012

[brb32804-bib-0016] Eyer, F. , Schuster, T. , Felgenhauer, N. , Pfab, R. , Strubel, T. , Saugel, B. , & Zilker, T. (2011). Risk assessment of moderate to severe alcohol withdrawal—predictors for seizures and delirium tremens in the course of withdrawal. Alcohol and Alcoholism, 46(4), 427–433. 21593124 10.1093/alcalc/agr053

[brb32804-bib-0017] Feige, B. , Scaal, S. , Hornyak, M. , Gann, H. , & Riemann, D. (2007). Sleep electroencephalographic spectral power after withdrawal from alcohol in alcohol‐dependent patients. Alcoholism: Clinical and Experimental Research, 33(1), 19–27. 10.1111/j.1530-0277.2006.00260.x 17207097

[brb32804-bib-0018] Ferguson, J. A. , Suelzer, C. J. , Eckert, G. J. , Zhou, X.‐H. , & Diffus, R. S. (1996). Risk factors for delirium tremens development. Journal of General Internal Medicine, 11(7), 410–414. 8842933 10.1007/BF02600188

[brb32804-bib-0019] Funderburk, W. (1949). Electroencephalographic studies in chronic alcoholism. Electroencephalography and Clinical Neurophysiology, 1, 369–370.

[brb32804-bib-0020] Gross, D. W. , & Gotman, J. (1999). Correlation of high‐frequency oscillations with the sleep–wake cycle and cognitive activity in humans. Neuroscience, 94(4), 1005–1018. 10625043 10.1016/s0306-4522(99)00343-7

[brb32804-bib-0021] Gruzelier, J. (2009). A theory of alpha/theta neurofeedback, creative performance enhancement, long distance functional connectivity and psychological integration. Cognitive Processing, 10(1), 101–109. 19082646 10.1007/s10339-008-0248-5

[brb32804-bib-0022] Hillbom, M. , Pieninkeroinen, I. , & Leone, M. (2003). Seizures in alcohol‐dependent patients. CNS Drugs, 17(14), 1013–1030. 14594442 10.2165/00023210-200317140-00002

[brb32804-bib-0023] Kaplan, R. F. , Glueck, B. C. , Hesselbrock, M. N. , & Reed Jr, H. (1985). Power and coherence analysis of the EEG in hospitalized alcoholics and nonalcoholic controls. Journal of Studies on Alcohol, 46(2), 122–127. 3990297 10.15288/jsa.1985.46.122

[brb32804-bib-0024] Klassen, B. T. , Hentz, J. G. , Shill, H. A. , Driver‐Dunckley, E. , Evidente, V. G. H. , Sabbagh, M. N. , Adler, C. H. , & Caviness, J. N. (2011). Quantitative EEG as a predictive biomarker for Parkinson disease dementia. Neurology, 77(2), 118–124. 21633128 10.1212/WNL.0b013e318224af8d PMC3140072

[brb32804-bib-0025] Ko, J. , Park, U. , Kim, D. , & Kang, S. W. (2021). Quantitative electroencephalogram standardization: A sex‐and age‐differentiated normative database. Frontiers in Neuroscience, 15, 766781. 34975376 10.3389/fnins.2021.766781 PMC8718919

[brb32804-bib-0026] Lee, J. H. , Jang, M. K. , Lee, J. Y. , Kim, S. M. , Kim, K. H. , Park, J. Y. , Lee, J. H. , Kim, H. Y. , & Yoo, J. Y. (2005). Clinical predictors for delirium tremens in alcohol dependence. Journal of Gastroenterology and Hepatology, 20(12), 1833–1837. 16336440 10.1111/j.1440-1746.2005.03932.x

[brb32804-bib-0027] Mainerova, B. , Prasko, J. , Latalova, K. , Axmann, K. , Cerna, M. , Horacek, R. , & Bradacova, R. (2015). Alcohol withdrawal delirium‐diagnosis, course and treatment. Biomedical Papers of the Medical Faculty of Palacky University in Olomouc , 159(1). 10.5507/bp.2013.089 24399242

[brb32804-bib-0028] Marrufo, M. V. , Vaquero, E. , Cardoso, M. A. J. , & Gomez, C. M. (2001). Temporal evolution of α and β bands during visual spatial attention. Cognitive Brain Research, 12(2), 315–320. 11587900 10.1016/s0926-6410(01)00025-8

[brb32804-bib-0029] Mennecier, D. , Thomas, M. , Arvers, P. , Corberand, D. , Sinayoko, L. , Bonnefoy, S. , Harnois, F. , & Thiolet, C. (2008). Factors predictive of complicated or severe alcohol withdrawal in alcohol dependent inpatients. Gastroentérologie clinique et biologique, 32(8‐9), 792–797. 18757147 10.1016/j.gcb.2008.06.004

[brb32804-bib-0030] Moselhy, H. F. , Georgiou, G. , & Kahn, A. (2001). Frontal lobe changes in alcoholism: A review of the literature. Alcohol and Alcoholism, 36(5), 357–368. 11524299 10.1093/alcalc/36.5.357

[brb32804-bib-0031] Nutt, D. (1999). Alcohol and the brain: Pharmacological insights for psychiatrists. The British Journal of Psychiatry, 175(2), 114–119. 10627792 10.1192/bjp.175.2.114

[brb32804-bib-0032] Nyhus, E. (2018). Brain networks related to beta oscillatory activity during episodic memory retrieval. Journal of Cognitive Neuroscience, 30(2), 174–187. 28984525 10.1162/jocn_a_01194

[brb32804-bib-0033] Odish, O. F. , Johnsen, K. , van Someren, P. , Roos, R. A. , & van Dijk, J. G. (2018). EEG may serve as a biomarker in Huntington's disease using machine learning automatic classification. Scientific Reports, 8(1), 1–8. 30382138 10.1038/s41598-018-34269-y PMC6208376

[brb32804-bib-0034] Park, S.‐P. , & Kwon, O.‐Y. (2009). Increased EEG current‐source density in the high Beta frequency band induced by levetiracetam adjunctive therapy in refractory partial epilepsy. Journal of Clinical Neurology, 5(4), 178–185. 20076799 10.3988/jcn.2009.5.4.178 PMC2806540

[brb32804-bib-0035] Paulucio, D. , da Costa, B. M. , Santos, C. G. , Velasques, B. , Ribeiro, P. , Gongora, M. , Cagy, M. , Alvarenga, R. L. , & Pompeu, F. A. (2017). Acute ethanol and taurine intake affect absolute alpha power in frontal cortex before and after exercise. Neuroscience Letters, 657, 5–10. 28743582 10.1016/j.neulet.2017.07.026

[brb32804-bib-0036] Pavlov, Y. G. , & Kotchoubey, B. (2017). EEG correlates of working memory performance in females. BMC Neuroscience, 18(1), 1–14. 28193169 10.1186/s12868-017-0344-5 PMC5307759

[brb32804-bib-0037] Poil, S.‐S. , De Haan, W. , Van Der Flier, W. M. , Mansvelder, H. D. , Scheltens, P. , & Linkenkaer‐Hansen, K. (2013). Integrative EEG biomarkers predict progression to Alzheimer's disease at the MCI stage. Frontiers in Aging Neuroscience, 5(58), 1–12. 24106478 10.3389/fnagi.2013.00058 PMC3789214

[brb32804-bib-0038] Rangaswamy, M. , Porjesz, B. , Chorlian, D. B. , Wang, K. , Jones, K. A. , Bauer, L. O. , Rohrbaugh, J. , O'connor, S. J. , Kuperman, S. , & Reich, T. (2002). Beta power in the EEG of alcoholics. Biological Psychiatry, 52(8), 831–842. 12372655 10.1016/s0006-3223(02)01362-8

[brb32804-bib-0039] Roberto, M. , & Varodaya, F. P. (2017). Synaptic targets: Chronic alcohol actions. Neuropharmacology, 1(122), 85–99. 10.1016/j.neuropharm.2017.01.013 PMC5479718 28108359

[brb32804-bib-0040] Sand, T. , Bråthen, G. , Michler, R. , Brodtkorb, E. , Helde, G. , & Bovim, G. (2002). Clinical utility of EEG in alcohol‐related seizures. Acta Neurologica Scandinavica, 105(1), 18–24. 11903104 10.1034/j.1600-0404.2002.00058.x

[brb32804-bib-0041] Shahin, A. J. , Picton, T. W. , & Miller, L. M. (2009). Brain oscillations during semantic evaluation of speech. Brain and Cognition, 70(3), 259–266. 19324486 10.1016/j.bandc.2009.02.008 PMC2683907

[brb32804-bib-0042] Trammell, J. P. , MacRae, P. G. , Davis, G. , Bergstedt, D. , & Anderson, A. E. (2017). The relationship of cognitive performance and the theta‐alpha power ratio is age‐dependent: An EEG study of short term memory and reasoning during task and resting‐state in healthy young and old adults. Frontiers in Aging Neuroscience, 9, 364. 29163144 10.3389/fnagi.2017.00364 PMC5682032

[brb32804-bib-0043] Tyler, L. K. , Marslen‐Wilson, W. D. , Randall, B. , Wright, P. , Devereux, B. J. , Zhuang, J. , Papoutsi, M. , & Stamatakis, E. A. (2011). Left inferior frontal cortex and syntax: Function, structure and behaviour in patients with left hemisphere damage. Brain, 134(2), 415–431. 21278407 10.1093/brain/awq369 PMC3030769

[brb32804-bib-0044] Van Der Kooi, A. W. , Zaal, I. J. , Klijn, F. A. , Koek, H. L. , Meijer, R. C. , Leijten, F. S. , & Slooter, A. J. (2015). Delirium detection using EEG. Chest, 147(1), 94–101. 25166725 10.1378/chest.13-3050

[brb32804-bib-0045] Weil, Z. M. , Corrigan, J. D. , & Karelina, K. (2018). Alcohol use disorder and traumatic brain injury. Alcohol Research: Current Reviews, 39(2), 171. 31198656 10.35946/arcr.v39.2.07 PMC6561403

[brb32804-bib-0046] Weiss, S. , & Mueller, H. M. (2012). “Too many betas do not spoil the broth”: The role of beta brain oscillations in language processing. Frontiers in Psychology, 3, 201. 22737138 10.3389/fpsyg.2012.00201 PMC3382410

[brb32804-bib-0047] Whittington, M. A. , Traub, R. D. , Kopell, N. , Ermentrout, B. , & Buhl, E. H. (2000). Inhibition‐based rhythms: Experimental and mathematical observations on network dynamics. International Journal of Psychophysiology, 38(3), 315–336. 11102670 10.1016/s0167-8760(00)00173-2

